# Supplementary figures and images for: Putting the microbiota to work: Epigenetic effects of early life antibiotic treatment are associated with immune-related pathways and reduced epithelial necrosis following Salmonella Typhimurium challenge in vitro
Source: PLoS One. 2020 Apr 27;15(4):e0231942. doi: 10.1371/journal.pone.0231942 (PMC7185588; doi:10.1371/journal.pone.0231942)

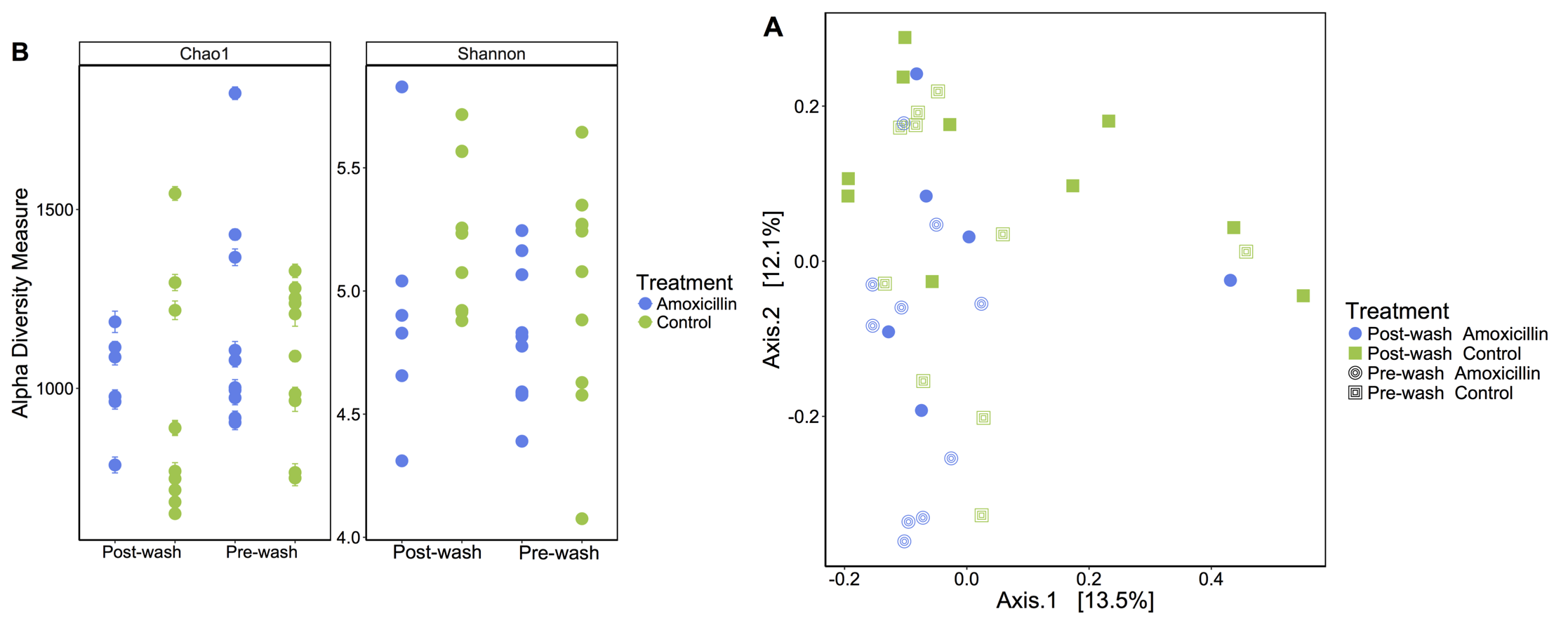

Supplement: S1 Fig — No distinctions were observed in microbial composition pre and post-washing of explants within each treatment as displayed by the indistinct clustering between pre and post wash within treatment groups (Amoxicillin and Control) in the principal coordinates plot of Bray Curtis dissimilarity (A). Alpha diversity did not differ with tissue washing within treatment groups (Amoxicillin and Control) as measured by Chao1 and Shannon Index (B). (TIFF) [file pone.0231942.s001.tiff]
