# Supplementary material for: Putting the microbiota to work: Epigenetic effects of early life antibiotic treatment are associated with immune-related pathways and reduced epithelial necrosis following Salmonella Typhimurium challenge in vitro
Source: PLoS One. 2020 Apr 27;15(4):e0231942. doi: 10.1371/journal.pone.0231942 (PMC7185588; doi:10.1371/journal.pone.0231942)
Supplement: Attachment — Submitted filename: plos one.docx [file pone.0231942.s004.docx]

Manuscript entitled “Putting the microbiota to work-------------- Salmonella Typhimurium challenge” submitted by Costa et al. deals with modulation of intestinal microbiome in piglets with antibiotic for 14 days and is of the opinion that antibiotic treatment in early life alters the gut microbiome resulting into decreased necrotic intestinal lesions following S. Typhimurium challenge. However, the proposed mechanism of gut modulation by antibiotic treatment in early phase of life cannot be correlated with the present study and their basis of discussion is purely hypothetical and is not supported with facts and findings.

**Specific comments**

1) Title needs to be changed
2) Abstract is not highlighting the important findings written properly.
3) Introduction is not written properly
4) No of replicates should be mentioned in material and methods
5) Transcriptomics data visualization is confusing
6) Data needs to be monitored statistically and carefully as in fig 1 the standard deviations too high
7) Figure no is not matching with text
8) Differential gene expression analysis are not appropriate in both the conditions.
9) Validation of RNA sequencing of data should have been supported by qPCR
10) Table 1 is very confusing. It is surprising to see the results of table 1 where authors have indicated down and up regulation of various biological processes. On the basis of which the authors have concluded the up regulation of immune response genes as the protective mechanism for intestinal pathogens. However, they have ignored the genes responsible for the basic physiology of living beings such as muscle contraction, heart contraction etc. that too are very significant in day to day life of living beings.
11) Manuscript is not discussed properly as the proposed modulatory mechanism is not clear.
12) Grammatically paper needs to be edited as English is very poor.

Overall, the manuscript is not acceptable.
