# Supplementary material for: Putting the microbiota to work: Epigenetic effects of early life antibiotic treatment are associated with immune-related pathways and reduced epithelial necrosis following Salmonella Typhimurium challenge in vitro
Source: PLoS One. 2020 Apr 27;15(4):e0231942. doi: 10.1371/journal.pone.0231942 (PMC7185588; doi:10.1371/journal.pone.0231942)
Supplement: Attachment — Submitted filename: plos one 2.docx [file pone.0231942.s005.docx]

Authors have tried to modify the manuscript entitled “Putting the microbiota to work-------------- Salmonella Typhimurium challenge in vitro” as per reviewer’s suggestions but it is still very confusing and not an exciting research paper. However, the proposed mechanism of gut modulation by antibiotic treatment in early phase of life cannot be correlated with the present study and their basis of discussion is still purely hypothetical.

**Author should clarify whether they have used prophylactic or metaphylactic studies. I still feel that the article is very confusing and is not acceptable for publication.**
