# Supplementary material for: Putting the microbiota to work: Epigenetic effects of early life antibiotic treatment are associated with immune-related pathways and reduced epithelial necrosis following Salmonella Typhimurium challenge in vitro
Source: PLoS One. 2020 Apr 27;15(4):e0231942. doi: 10.1371/journal.pone.0231942 (PMC7185588; doi:10.1371/journal.pone.0231942)
Supplement: Attachment — Submitted filename: Reviewer_comments.docx [file pone.0231942.s006.docx]

Reviewer #1: Authors have tried to modify the manuscript entitled “Putting the microbiota to work-------------- Salmonella Typhimurium challenge in vitro” as per reviewer’s suggestions but it is still very confusing and not an exciting research paper. However, the proposed mechanism of gut modulation by antibiotic treatment in early phase of life cannot be correlated with the present study and their basis of discussion is still purely hypothetical.
Author should clarify whether they have used prophylactic or metaphylactic studies. I still feel that the article is very confusing and is not acceptable for publication.

Respectfully, we are unsure on how to interpret these comments. This study is not a prophylactic or a metaphylactic study. Here, we described the concept of exploiting the gut microbiome as a “innate immunity” mechanism, mitigating lesions associated with *Salmonella*.

Thus, we were unable to identify any suggestions or alternatives to improve the manuscript based on the reviewer’s notes. The reviewer suggests that the text is “confusing” and “not exciting” without giving specific examples of what should be improved, using vague statements such as “*I still feel that the article is very confusing*”.

Unfortunately, we cannot address any comments because there were no effective comments to be addressed.

Reviewer #2: In the revised version of the manuscript, the authors have attempted to address many of the concerns raised during review of the initial submission. The manuscript will benefit if the authors could further elaborate on the below mentioned aspects:

- Lines 390-391. “These effects, however, should be extrapolated cautiously because changes in the microbiota are suggested to be antibiotic-specific”. As mentioned earlier, this statement needed to be cited with appropriate examples of antibiotic classes and the resulting changes in microbiota. In the revised manuscript, the authors have only included a reference for this statement; instead a couple of lines to explain this association, along with specific examples of antibiotic classes and the microbiota impacted, would be beneficial for the readers.

We have taken the reviewer’s suggestion into consideration and improved this section, which can be seen in the revised version of the manuscript between lines 390-398.

- In continuation of my previous comments, can the authors propose (at least in the discussion) any alternate strategy to modulate the normal flora for the observed immunostimulatory effect without the use of antibiotics? This point remains a concern because as mentioned earlier (and agreed by the authors), antibiotic prophylaxis is not a viable option. The entire study is based on the impact of amoxicillin on gut microbiota, immune response, and subsequent Salmonella infection. The authors state “the goal is to reduce the use of antibiotics by developing novel methods to modulate the microbiome. The development of such methods is not the goal of this study. .... we do provide here the basis and justification for such studies, the concept that microbiome modulation can affect infection outcomes.” However, the concept that modulation of the microbiome can affect infection outcomes is already known. To me, the study attempts to address a mechanism for the observed effects but as the experimental layout used (amoxicillin exposure) would not be a feasible option, the manuscript will be more enriching if alternate ways to achieve this effect can at least be proposed/hypothesized.

We appreciate the reviewer’s concern and agree that, without the proper context, findings from this study could be erroneously interpreted. Thus, we have further disserted about this issue by proposing possible alternatives to the use of antibiotics to modulate the gut microbiome, including successful cases of such approach. You may find this discussion between lines 418-427 in the revised version of the manuscript.
